# Tuberculosis and poverty: the contribution of patient costs in sub-Saharan Africa – a systematic review

**DOI:** 10.1186/1471-2458-12-980

**Published:** 2012-11-14

**Authors:** Devra M Barter, Stephen O Agboola, Megan B Murray, Till Bärnighausen

**Affiliations:** 1 Department of Global Health and Population, Harvard School of Public Health, 677 Huntington Avenue, Boston, MA 02115, USA; 2 Department of Epidemiology, Harvard School of Public Health, 677 Huntington Avenue, Boston, MA 02115, USA; 3 Africa Centre for Health and Population Studies, University of KwaZulu-Natal, KwaZulu-Natal, Mtubatuba, South Africa

**Keywords:** Tuberculosis, Economic impact, Out-of-pocket costs, Africa

## Abstract

**Background:**

Tuberculosis (TB) is known to disproportionately affect the most economically disadvantaged strata of society. Many studies have assessed the association between poverty and TB, but only a few have assessed the direct financial burden TB treatment and care can place on households. Patient costs can be particularly burdensome for TB-affected households in sub-Saharan Africa where poverty levels are high; these costs include the direct costs of medical and non-medical expenditures and the indirect costs of time utilizing healthcare or lost wages. In order to comprehensively assess the existing evidence on the costs that TB patients incur, we undertook a systematic review of the literature.

**Methods:**

PubMed, EMBASE, Science Citation Index, Social Science Citation Index, EconLit, Dissertation Abstracts, CINAHL, and Sociological Abstracts databases were searched, and 5,114 articles were identified. Articles were included in the final review if they contained a quantitative measure of direct or indirect patient costs for treatment or care for pulmonary TB in sub-Saharan Africa and were published from January 1, 1994 to Dec 31, 2010. Cost data were extracted from each study and converted to 2010 international dollars (I$).

**Results:**

Thirty articles met all of the inclusion criteria. Twenty-one studies reported both direct and indirect costs; eight studies reported only direct costs; and one study reported only indirect costs. Depending on type of costs, costs varied from less than I$1 to almost I$600 or from a small fraction of mean monthly income for average annual income earners to over 10 times average annual income for income earners in the income-poorest 20% of the population. Out of the eleven types of TB patient costs identified in this review, the costs for hospitalization, medication, transportation, and care in the private sector were largest.

**Conclusion:**

TB patients and households in sub-Saharan Africa often incurred high costs when utilizing TB treatment and care, both within and outside of Directly Observed Therapy Short-course (DOTS) programs. For many households, TB treatment and care-related costs were considered to be catastrophic because the patient costs incurred commonly amounted to 10% or more of per capita incomes in the countries where the primary studies included in this review were conducted. Our results suggest that policies to decrease direct and indirect TB patient costs are urgently needed to prevent poverty due to TB treatment and care for those affected by the disease.

## Background

In 2009, tuberculosis (TB) was the world’s 7th leading cause of death, resulting in 1.7 million deaths worldwide, more than 9.4 million new infections and 14 million prevalent cases [1]. TB is often known as “a disease of the poor” because the burden of TB follows a strong socioeconomic gradient both between and within countries, and also within the poorest communities of countries with high TB incidence [2]. Some studies have shown a strong association between poverty and TB and have demonstrated that poor and vulnerable groups are at an increased risk of TB infection, have a higher prevalence of disease, have worse outcomes (including mortality), and display worse TB care-seeking behaviors [3-8]. Risk factors for these TB-related outcomes include structures, behaviors and other diseases commonly associated with poverty - overcrowded living or working conditions, poor nutrition, smoking, alcoholism, diabetes, exposure to indoor air pollution and HIV [2,7-10].

It is also well-known that TB can contribute to poverty by reducing patients’ physical strength and ability to work [8,11-13]. However, another pathway through which TB can affect households’ economic situation, the costs patients incur when utilizing TB care, has been less studied. These costs include both direct out-of-pocket costs incurred when seeking treatment and care and the indirect, or time costs, associated with utilizing healthcare. While most countries with high TB burden provide free sputum smear microscopy for patients with suspected pulmonary TB, more than half of these 22 countries charge for other TB-related diagnostic tests such as radiography, sputum culture, and drug-susceptibility testing [14]. Under Directly Observed Therapy Short-course (DOTS) programs, all high burden TB countries provide free first line anti-TB medication, but many patients purchase anti-TB drugs in private pharmacies (some without prescriptions), which can be costly [14,15]. In high TB burden countries, 60% of overall health expenditure is in the private sector, and a large proportion of these expenditures are paid out-of-pocket by patients [14].

A number of previous studies have documented the downstream consequences of the direct and indirect costs that TB patients incur. More than 50% of TB patients have been reported to experience financial difficulties due to TB [16], and these costs can be “catastrophic” in that they amount to more than 10% of patients’ or households’ annual income [17-19]. TB patient costs have been shown to lead to reduced food consumption, diversion of resources from other types of healthcare, taking children out of school, and borrowing or selling assets [17,19-21]. Furthermore, financial constraints have been shown to predict non-adherence to TB medication [16]. In general, the World Health Organization (WHO) estimates that 100 million people every year fall into poverty from paying for health services [22].

One earlier review reports on the overall costs TB-patients in Africa face during the pre- and post-diagnosis phases of TB treatment and care as well as coping mechanisms for catastrophic costs [23]. In this study, we expand on this previous assessment by broadening the evidence base on TB patient costs in sub-Saharan Africa through screening of additional databases and broadening the study design inclusion criteria, systematically identifying the particular types of TB patient costs (both direct and indirect), systematically reviewing the evidence on the cost quantities for each cost type, and providing benchmarks for the magnitude of cost burdens on TB patients and households.

## Methods

### Data sources and search strategies

We used eight electronic databases to identify papers reporting on patient costs for TB care in sub-Saharan Africa available by January 3-4, 2011: PubMed, Embase, Science Citation Index, Social Science Citation Index, EconLit, Dissertation Abstracts, Cumulative Index to Nursing and Allied Health Literature (CINAHL), and Sociological Abstracts. Each search strategy comprised a Boolean operator of “and” with two elements: tuberculosis and cost/economic aspects. For the PubMed search, MeSH and “all fields” terms comprising tuberculosis and OR fields for cost estimates such as “employment,” “out of pocket,” “patient costs” and MeSH terms for “costs and cost analysis” were used. Similar search strategies were employed for the other 7 databases (see Additional file 1 for the precise search algorithms for each database). Each database was searched from the earliest referenced publication date through January 1, 2011. Studies were included regardless of language. Two reviewers independently screened articles identified from the initial search of the databases by title and/or abstract.

To identify additional articles, conference abstracts written in English from 1994-2010 from the International Union Against Tuberculosis and Lung Disease (IUATLD) annual conference were searched. Furthermore, we performed a secondary search of reference lists of articles identified through the database search, including both the primary studies included in our synthesis and review studies.

Articles were considered for inclusion if they contained a quantitative measure of a direct or indirect patient-incurred cost (including time costs) relating to TB treatment or care for adult pulmonary tuberculosis. Following Rajeswari et al. [24] and Jackson et al. [25] we defined costs as follows: Direct costs included both medical expenditures (such as consultation fees or costs of medication or diagnostic tests) and non-medical expenditures (such as money spent on travel, lodging, and food for both patients and caregivers). Indirect costs were defined as time costs associated with utilizing healthcare, or time costs converted into monetary units based on loss of wages for both patients and caregivers or decreased earning ability [26].

We excluded the following articles: i) published before 1994 when DOTS was officially launched as a framework for a TB control strategy recommended by the WHO [27]; ii) not taking place in Sub-Saharan Africa; iii) not pertaining to TB; iv) not involving human subjects; v) on MDR-TB, HIV/TB co-infection, latent or pediatric TB; vi) focusing solely on diagnostic tests, screening tests, or vaccinations; vii ) not containing any primary data on cost estimates or economic analysis; or viii) not relating to individual patient costs.

Articles were assessed for study quality. In particular, we examined the studies to ensure that we included only those in our final review that had clearly defined objectives, clearly defined study populations, and a quantitative measure of patient-costs. Additionally, articles were categorized by study type and whether costs were incurred pre- or post-diagnosis. We adhered to the PRISMA guidelines [28].

### Data extraction and analysis

In addition to study-specific variables (authors, study type, year, location, setting, period of observation, population under study, and study objectives), the two reviewers extracted patient-borne quantitative direct and indirect cost measurements. We extracted only costs measured empirically in the reviewed studies. Costs that were extrapolated or projected in mathematical models were not included in the analysis. Data were categorized into costs related to health insurance, prepayment, consultation or provider fees, hospitalization, medication, and diagnostic test costs, traditional healer and food costs, travel costs, time costs, reported impact on income, reported direct, indirect, and total costs, caregiver costs and catastrophic costs.

To compare costs expressed in different currencies and measured in different years, we converted all cost 180 estimates into 2010 international dollars (I$). We rounded all cost estimates to the nearest integer except for costs less than 1I$, which we rounded to the second decimal place. For costs that were presented in US$, costs were first converted to respective local currency units using OANDA currency conversions [29] based on exchange rates at the commencement of the study period. For studies that did not specify the year or period of currency estimates, January 1^st^ of the beginning of the study year was used as a standard conversion date except for one study in Botswana [30], for which conversion rates were only available beginning November 1, 1993 instead of January 1, 1993, and for one study in Uganda [31], for which conversion rates were available beginning January 1, 1996 instead of January 1, 1992. Next, local currency units were adjusted to 2010 rates using the International Monetary Fund’s database on average consumer price inflation over time [32]. Finally, costs were adjusted to 2010 international dollars based on the World Bank’s purchasing power parity (PPP) conversion factors (in local currency units per international dollar) [33].

To compare costs across studies, we report travel costs as single visit costs and hospitalization costs over the entire treatment period. To provide a benchmark for the magnitude of cost burdens of TB care on patients, we expressed the expenditures as percentage of per-capita annual (or monthly) GDP (in I$) of the country and in the year when the study, which generated the cost estimates, was conducted. The per-capita GDP figures are taken from the *World Development Indicators* published by the World Bank [33]. Per-capita GDP, i.e., the average income, is one benchmark that is meaningful to understand and commonly used for such purposes, and we thus use it here. However, since TB is a disease that predominantly affects poorer populations, we also express the cost estimates as a percentage of an alternative income benchmark—the per-capita income of the income-poorest 20% of the population, calculated according to the following equation:

(1) $$\frac{\text{Total GDP}*\text{Income share of the income}\text{-}\text{poorest}\;20\%\;\text{of the population}}{\text{Total population size}*0.2}$$

We chose these two income benchmarks, rather than study population-specific incomes, because very few of the studies included in our review reported the study population income. We also report whether the patient costs of TB treatment are “catastrophic” for the person of average income or the person of average income amongst the income-poorest 20% of the population, classifying costs as “catastrophic” when they were at least 10% of average annual income for the respective population. While definitions of catastrophic expenditures commonly relate to household income, [18,34-37] we chose to use 10% of annual individual income as a benchmark for catastrophic costs because for most studies we lack household-level income data as well as the household-level TB data that would be necessary to judge whether the financial burdens of TB care and treatment is “catastrophic” or not. Without the latter data, household-level income data is not an appropriate indicator since TB tends to cluster in households [38-40].

## Results

**Figure 1:**
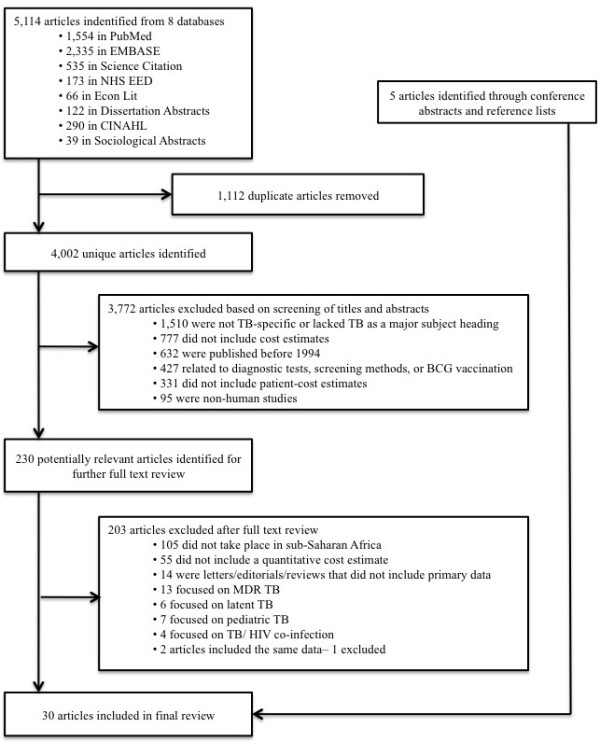
Flowchart of the systematic review.

5,114 articles were identified from the initial search of the eight databases. After excluding 1,112 duplicate articles, 1,510 were excluded because they did not include TB as a major subject heading, 777 did not include cost estimates, 632 were published before 1994, 427 focused solely on diagnostic tests, screening tests or vaccinations, 331 did not include patient cost estimates, and 95 did not involve human subjects. Reviewing the full text of the remaining 230 articles, we found that 55 did not include a quantitative cost measurement; 14 were reviews, commentaries, letters or editorials that did not include primary data; 7 were on pediatric TB, 13 on MDR-TB, 6 on latent TB, 4 on HIV/TB, 105 studies did not take place in sub-Saharan Africa; and 2 studies were by the same authors and included the same data (leading to the exclusion of 1 of the 2 studies). This selection process resulted in 25 relevant studies; 5 additional relevant publications were identified through the search of the conference database and reference lists, so that a total of 30 articles were included in the final synthesis of our review (see Figure 1).

Table 1 describes each of the 30 studies in terms of their study populations, main objectives, types of cost estimates, and time period in which costs were incurred (pre- vs. post-diagnosis). Eight studies reported direct costs, one study reported indirect costs, and twenty-one studies reported both indirect and direct costs. Table 2 describes the cost categories, including definitions for each cost type, whether the costs are considered direct or indirect, whether the cost are incurred pre- or post-diagnosis, the number of studies reporting a particular cost type, and the cost range and median among all studies reporting the cost type.

**Table 1:** Summary of Studies

**Author (Year)**	**Country**	**Type of study**	**Population under study**	**Primary objectives**	**Types of costs reported**	**Time period of costs (Pre vs. post diagnosis)**
Aspler, et al. (1998) [41]	Zambia	Cross-sectional	103 patients aged ⩾18 years with active or extra-pulmonary TB who had been on treatment for 6-10 weeks	To estimate TB patient costs for treatment and diagnosis and cost determinants	Pre-diagnosis, treatment, time, travel, medication, consultation, hospitalization, food, health insurance, and diagnostic test costs	Both
Awofeso, N. (1998) [42]	Nigeria	Prospective cohort	2144 symptomatic smear-positive patients in two study periods	To discuss the implications of pre-payment versus free medication therapy on treatment and case-finding of TB patients	Medication costs	Post-diagnosis
Bevan, E. (1997) [43]	Kenya	Unknown	Unknown	Letter to describe other costs associated with DOTS	Daily inpatient care, travel, and other medical expenses	Post-diagnosis
Brouwer, et al. (1998) [44]	Malawi	Cross-sectional	89 smear-positive pulmonary TB patients admitted to Queen Elizabeth Central Hospital	To investigate how TB patients utilize traditional healers and traditional medicine in their care-seeking behaviors	Total fixed and variable costs, time, and traditional healer costs	Pre-diagnosis
Cambanis, et al. (2005) [45]	Ethiopia	Cross-sectional	243 patients undergoing sputum examination for TB diagnosis	To assess factors related to patient delay in presenting to health services for the diagnosis of TB	Time and travel costs	Pre-diagnosis
Chard, S. (2001) [46]	Uganda	Cross-sectional	89 female patients aged ⩾18 years identified from a TB clinic	To examine treatment seeking, health beliefs, and social networks of female Ugandan TB patients	Time, travel, medication, traditional healers, and costs for “tipping” healthcare providers	Both
Chard, S. (2009) [47]	Uganda	Cross-sectional	65 women aged ⩾18 years with a diagnosis of pulmonary TB, and receiving outpatient TB treatment from one of three TB clinics	To explore the TB treatment-seeking process of Ugandan women in order to determine the routes to effective government TB treatment	Private providers and traditional healer costs	Both
Datiko and Lindtjørn (2010) [48]	Ethiopia	Cost-effectiveness analysis	229 smear-positive patients	To determine the cost and cost-effectiveness of involving health extension workers in TB treatment under a community-based model	Time, caregiver, food, direct, and total costs	Post-diagnosis
Edginton, et al. (2002) [49]	South Africa	Qualitative	114 hospital TB patients and 75 clinic TB patients and community members were interviewed	To assess the beliefs and experiences about TB from the perspective of patients and community members in order to assess the impact of presentation to health services and treatment adherence	Time and travel costs	Post-diagnosis
Floyd, et al. (2003) [50]	Malawi	Cost-effectiveness analysis	2,174 new smear-positive and -negative patients registered for treatment in 1997; 2,821 new smear-positive and -negative patients registered for treatment in 1998	To assess the cost and cost-effectiveness of new treatment strategies for new pulmonary TB patients introduced in Malawi in 1997	Time, travel, hospitalization, caregiver, and DOTS costs	Post-diagnosis
Floyd, et al. (1997) [51]	South Africa	Cost-effectiveness analysis	New smear-positive adult patients	To conduct an economic evaluation of directly observed treatment and conventionally delivered treatment for the management of new adult TB cases	Time, travel, hospitalization, total, and DOTS costs	Post-diagnosis
Gibson, et al. (1998) [52]	Sierra Leone	Cross-sectional	54 inpatients, 18 outpatients, and 17 staff members in 6 TB Centers	To evaluate the impact of patient poverty and staff salaries on patient costs for TB treatment within a sub-national TB program	Pre-program, program time, and total costs	Both
Harper, et al. (2003) [53]	The Gambia	Qualitative	443 patients and clinic staff participated in focus groups, in-depth interviews, and semi-structured interviews	To evaluate the factors related to shortages of case tracing and adherence to treatment using qualitative methods with a cohort of TB patients	Travel and private treatment costs	Both
Kemp, et al. (2007) [54]	Malawi	Cross-sectional	179 smear-positive and -negative TB patients who were in the intensive phase of treatment	To assess the relative costs of accessing a TB diagnosis for the poor and for women in urban Lilongwe, Malawi, where public health services are accessible within 6km and are provided free of charge	Time, travel, medication, and food costs	Both
Mesfin, et al. (2010) [55]	Ethiopia	Prospective cohort	537 newly diagnosed smear-positive pulmonary TB patients and 387 newly diagnosed smear-negative pulmonary TB patients ≥15	To investigate costs of TB diagnosis incurred by patients, their escorts, and the public health system in 10 districts in Ethiopia	Caregiver, time, travel, medication, consultation, hospital admission, and lodging costs	Both
Moalosi, et al. (2003) [56]	Botswana	Cost-effectiveness analysis	50 caregivers of TB patients on home-based care	To determine the affordability and cost-effectiveness of home-based DOTS vs. hospital-based DOTS for TB patients and to describe the characteristics of patients and their caregivers	Total, time, travel, medication and hospitalization costs for caregivers	Both
Needham, et al. (1996) [57]	Zambia	Cross-sectional	23 adult inpatients and outpatients with a diagnosis of pulmonary TB	Letter in response to Pocock et al. 1996 to assess patient-related economic barriers to TB diagnosis in Lusaka, Zambia	Medical, non-medical, time, and caregiver costs	Both
Needham, et al. (1998) [58]	Zambia	Cross-sectional	202 adult inpatients and outpatients registering with new pulmonary TB at the Chest Clinic	To study the pre-diagnosis economic impact burden and barrers to care seeking for TB patients in urban Zambia	Time, travel, consultation, caregiver, private provider, traditional healer, insurance, diagnostic, treatment, and food costs	Both
Needham, et al. (2004) [59]	Zambia	Qualitative	202 adult patients with pulmonary tuberculosis	To assess the barriers to successful care seeking faced by TB patients in urban Zambia	Time, travel, caregiver, and government health insurance costs	Pre-diagnosis
Nganda, et al. (2003) [60]	Kenya	Cost-effectiveness analysis	New smear-positive, new smear-negative and extra-pulmonary adult patients; for each type of patient, two alternative approaches to treatment were evaluated: the conventional approach used until September 1997 and the new approach introduced in October 1997	To assess the cost and cost-effectiveness of new treatment strategies, involving decentralization of care from hospitals to peripheral health facilities and the community, compared to the conventional approaches used until October 1997	Total, travel, hospitalization, TB clinic, and DOTS costs	Post-diagnosis
Okello, et al. (2003) [61]	Uganda	Cost-effectiveness analysis	New smear-positive pulmonary patients under two strategies: the conventional hospital-based approach used from 1995 thorough 1997, and the new community-based approach introduced in 1998	To assess the cost and cost-effectiveness of conventional hospital-based care with the new community-based care for new smear-positive pulmonary TB patients	Time, travel, hospitalization, and total DOTS costs	Post-diagnosis
Pocock, et al. (1996) [62]	Malawi	Cross-sectional	100 adult patients with smear-positive and extrapulmonary TB admitted to the TB ward, Queen Elizabeth Central Hospital, for 2 months of treatment	Letter investigating impacts of long hospitalization from the patients’ perspective	Time costs	Post-diagnosis
Saunderson, P.R. (1995) [31]	Uganda	Cost-effectiveness analysis	34 patients attending a hospital run by a non-governmental organization	To analyze the costs and cost-effectiveness of the current TB control strategy and an alternative ambulatory treatment strategy	Total, time, hospitalization, and pre-diagnosis costs	Both
Sinanovic, et al. (2003) [63]	South Africa	Cost-effectiveness analysis	New smear-positive and retreatment pulmonary TB patients started on treatment in two townships of Metropolitan Cape Town (Guguletu, where both clinic and community care were provided, and Nyanga, whereonly clinic-based care was provided)	To evaluate the affordability and cost-effectiveness of community involvement in TB care	Total, time, and travel costs	Post-diagnosis
Sinanovic and Kumaranay-ake (2006) [64]	South Africa	Cost-effectiveness analysis	1,182 new sputum positive patients at 2 public-private workplace sites (PWP), 2 public-non-governmental organization partnership sites (PNP) and 2 purely public sites	To estimate the cost and cost-effectiveness of different types of public-private-partnerships in TB treatment and the financing required for the different models from the provincial TB program from the patient and provider perspective	Total, time, and travel costs	Post-diagnosis
Steen and Mazonde (1999) [30]	Botswana	Cross-sectional	212 New and retreated patients with smear-positive pulmonary TB	To estimate the health-seeking behaviors of TB patients and their beliefs and attitudes of the disease	Outpatient fees	Post-diagnosis
Vassall, et al. (2010) [65]	Ethiopia	Cross-sectional	250 patients ⩾ 15 years using TB-HIV pilot services and diagnosed with and being treated for TB, HIV, or both	To measure patients costs of TB-HIV services from hospital-based pilot sites for collaborative TB-HIV interventions	Direct, indirect, transport, total	Both
Wandwalo, et al. (2005) [66]	Tanzania	Cost-effectiveness analysis	42 treatment supervisors and 103 new smear-positive, smear-negative, and extrapulmonary TB patients 5 years	To determine the cost and cost-effectiveness of community-based DOTS versus health facility treatment of TB in urban Tanzania	Direct, indirect, time, and total costs	Post-diagnosis
Wilkinson, et al. (1997) [67]	South Africa	Cost-effectiveness analysis	TB patients under the Hlabisa strategy (1991-preent), the former Hlabisa strategy (until 1991), the Department of Health strategy, and the SANTA strategy based on sanatorium care	To conduct an economic analysis of the Hlabisa community-based DOTS management compared to three alternative strategies	Total, hospitalization, and travel costs	Post-diagnosis
Wyss, et al. (2001) [68]	Tanzania	Cross-sectional	191 TB cases in 3 surveillance areas who had smear-positive, extrapulmonary, or relapse TB	To assess household level costs of TB and to compare them with provider costs of the National TB Control Program	Diagnostic test, time, traditional healer, private provider, hospitalization, caregiver, and travel costs	Both

**Table 2:** Types of Costs

**Cost categories**	**Definition**	**Direct or indirect**	**Pre- or post-diagnosis**	**Number of studies reporting cost category**	**Range (median) of costs**
Health insurance	Costs required for national health insurance schemes to finance TB care	Direct	Pre-diagnosis	2	I$2- I$3 (I$2)
Consultation or prepayment fees	Costs charged by providers before diagnosis or treatment	Direct	Pre-diagnosis	4	I$2- I$7 (I$3)
Private provider fees	Costs charged in the private sector rather than the public sector	Direct	Both	4	I$24- I$141 (I$41)
Hospitalization	Costs associated with hospitalization due to TB	Direct	Post-diagnosis	10	I$1- I$530 (I$80)
Medication	Costs of medications including standard TB treatment under non-DOTS systems and other drugs	Direct	Post-diagnosis	5	I$15- I$548 (I$21)^a^
Diagnostic tests	Costs for tests other than sputum microscopy such as x-rays, chest radiographs, or other laboratory tests	Direct	Pre-diagnosis	3	I$7- I$10 (I$9)
Traditional healer	Costs associated with seeking traditional healers before Western medical care	Direct	Pre-diagnosis	5	I$4- I$563 (I$15)
Food	Costs for regular food and food separate from normal diets such as potatoes, eggs, meat, fruit, and soft drinks [58]	Direct	Post-diagnosis	4	I$4- I$36 (I$10)
Travel	Costs for travel association with pre-diagnosis, consultation, diagnosis, treatment, pill collection, DOTS and follow-up treatment visits.	Direct	Both	18	I$0.17- I$70 (I$5)
Time	Time and indirect costs associated with time spent seeking/receiving care and lost work time	Indirect	Both	21	I$0.23- I$412 (I$16)^b^
Caregiver	Costs to those accompanying patients to TB care visits, retrieving medications on their behalf, or cost of care-giving activities. Direct costs encompass travel expenses, food, or other costs such as paying for an overnight stay when making a long journey. Indirect costs include loss of income and time spent accompanying patients or providing care-giving activities.	Both	Both	8	I$0.41- I$1,510 (I$11)^c^

^a^ Note: Some medication estimates also include the cost of user/consultation fees; ^b^ Costs include only reported costs of income lost due to time; ^c^ Based on different categories of costs.

### Health insurance, prepayment fees, consultation and private provider fees

Two studies reported health insurance fees that ranged from I$2 to I$3 in Zambia [41,59], and four studies reported consultation or prepayment fees that ranged from I$2 in Ethiopia [55] and Botswana [30] to I$7 in Zambia [58]. Patients who did not seek care from the public sector paid fees for care in the private sector. Four studies noted such fees for private services, which ranged from I$24 (median I$10) in Zambia [58] to I$141 in Uganda [47] (see Table 3). Additionally, one study from Uganda reported the practice of “tipping” healthcare providers in the range of I$5- I$40 [46].

**Table 3:** Health insurance, consultation/prepayment fees and private provider fees

**Author(s) (year)**	**Country**	**Cost estimate (I$)**	**% Annual per-capita income (entire population)**	**% Annual per-capita income (income-poorest 20% of the population)**	**Notes**
**Health insurance costs**					
Aspler, et al. [41]	Zambia	2	0.43	2	67% of patients reported paying median health insurance user fees (IQR I$1.79- I$1.97)
Needham, et al. [59]	Zambia	3	0.69	3^a^	Mean monthly fees for government-sponsored health insurance (range I$2-I$3)
**Consultation/prepayment fees**					
Aspler, et al. [41]	Zambia	4	0.78	4	Median one time consultation fee (IQR I$4- I$7)
Mesfin, et al. [55]	Ethiopia	2	0.93	2	Mean consultation fees per visit (median I$0)
Needham, et al. [58]	Zambia	7	2	10	Mean one-time consultation fees (median I$8)
Steen and Masonde [30]	Botswana	2	0.06	0.43^b^	One-time prepayment outpatient fee
**Private provider fees**					
Chard, S. [47]	Uganda	141	47	154^c^	Private clinic treatment costs
Harper, et al. [53]	The Gambia	44	11	54^d^	Costs spent on private treatment
Needham, et al. [58]	Zambia	24	6	35	Mean costs to see a private physician (median I$15)
Wyss, et al. [68]	Tanzania	38	12	29^e^	Unit cost for private services

^a^ Income share based on 1996 estimates instead of 1995; ^b^ Income share based on 1994 estimates instead of 1993; ^c^ Income share based on 1999 estimates instead of 1998; ^d^ Income share based on 1998 estimates instead of 2000; ^e^ Income share based on 2000 estimates instead of 1996.

### Hospitalization, medication, and diagnostic tests costs

Ten studies reported hospitalization expenses. Costs ranged from I$4 in Uganda [61] to over I$530 in Kenya [60]. Some patients were required to pay hospital admission fees. Patients in Freetown, Sierra Leone paid an average of I$1 at a missionary hospital and I$47 at a government hospital, which included the cost of food [52]. Five studies reported medication costs that ranged from I$20 in Uganda [46] to I$548 in Nigeria [42] (see Table 4). Moreover, one study from Kenya reported that patients paid I$46 monthly for syringes and needles for streptomycin treatment (not including streptomycin itself) [43]. Three studies reported diagnostic test costs other than sputum smears which ranged from I$7 for chest radiographs [41] to I$10 for examination, laboratory, and X-ray fees in Tanzania [68].

**Table 4:** Hospitalization, medication, and diagnostic test costs

**Author(s) (year)**	**Country**	**Cost estimate (I$)**	**% Annual per-capita income (entire population)**	**% Annual per-capita income (income-poorest 20% of the population)**	**Notes**
**Hospitalization costs**					
Aspler, et al. [41]	Zambia	14	3	16	Median costs (IQR I$4- I$19)
Floyd, et al. [51]	South Africa	119	3	17^a^	Mean cost of 18-day hospital stay under DOTS (I$7 per day)
Floyd, et al. [51]	South Africa	407	11	59^a^	Mean cost of 60-day hospital stay under conventional system (I$7/day)
Floyd, et al. [50]	Malawi	498	262	1048	Mean cost of 58-day hospital stay under hospital-based strategy for smear-positive patients (I$9/day)
Floyd, et al. [50]	Malawi	138	73	289	Mean cost of 16-day hospital stay under community-based DOTS strategy for smear-positive patients (I$9/day)
Floyd, et al. [50]	Malawi	32	17	66	Mean cost of 8-day hospital stay under hospital-based and community-based DOTS strategies for smear-negative patients (I$9/day)
Gibson and Boillot [52]	Sierra Leone	1	0.53	2^b^	Mean hospital admission fees at a missionary hospital
Gibson and Boillot [52]	Sierra Leone	47	18	58^b^	Mean hospital admission fees at a government hospital
Mesfin, et al. [55]	Ethiopia	4	2	5	Mean cost of hospital admissions (median I$0)
Nganda, et al. [60]	Kenya	530	101	336^c^	Mean cost of 60-day hospital stay under hospital-based system for smear-positive patients (I$9/ day) (96% CI I$5- I$13)
Nganda, et al. [60]	Kenya	34	7	22^c^	Mean cost of 4-day hospital stay under community-based DOTS for smear-positive patients (I$9/ day) (96% CI I$5- I$13)
Okello, et al. [61]	Uganda	219	73	240^d^	Mean cost of 60-day hospital stay under conventional hospital-based care strategy for smear-positive patients (I$4/ day)
Okello, et al. [61]	Uganda	70	24	77^d^	Mean cost of 19-day hospital stay under community-based care strategy for smear-positive patients (I$4/ day)
Saunderson, P. [31]	Uganda	91	39	126	Mean cost for a 2-month hospital stay
Wilkinson, et al. [67]	South Africa	139	3.62	20^e^	Mean cost of 17.5-day hospital stay under community-based DOTS strategy (I$8/ day)
Wyss et al. [68]	Tanzania	15	5	12^f^	Hospitalization costs reported for one month
**Medication costs**					
Aspler, et al. [41]	Zambia	15	3	18	Median costs for additional medications (IQR I$9- I$21)
Awofeso, N. [42]	Nigeria	548^g^	121	589^h^	Mid-range, one-time medication costs (range I$199- I$897)^g^
Chard, S. [46]	Uganda	20	7	22^i^	Mean costs for medications (range I$4- I$37)
Kemp, et al. [54]	Malawi	50	27	106^j^	Mean costs for smear-negative patients for user fees and drug costs outside of government health facilities (median I$19)
Kemp, et al. [54]	Malawi	18	9	37^j^	Mean costs for smear-positive patients for user fees and drug costs outside of government health facilities (median I$6)
Mesfin, et al. [55]	Ethiopia	22	12	25	Mean costs for additional medications (median I$7)
**Diagnostic test costs**					
Aspler, et al. [41]	Zambia	7	2	8	Median cost for chest radiographic (IQR I$4-I$7)
Needham, et al. [58]	Zambia	9	2	13	Mean cost for diagnostic tests (unspecified) (median I$13)
Wyss, et al. [68]	Tanzania	10	3	7^f^	Unit cost for examination, laboratory, and X-rays

^a^ Income share based on 1995 estimates instead of 1994; ^b^ Income share assumed to be 6% by authors in absence of World Bank data; ^c^ Income share based on 1997 estimates instead of 1998; ^d^ Income share based on 1999 estimates instead of 1998; ^e^ Income share based on 1995 estimates instead of 1996; ^f^ Income share based on 2000 estimates instead of 1996; ^g^ Note: the study listed these costs as “prepayment fees,” but the costs were actually for medications. Moreover, these medication costs are reported with imprecision (i.e., a wide range), weakening the strength of the conclusion that TB medication costs were high in Nigeria; ^h^ Income share based on 1992 estimates instead of 1993; ^i^ Income share based on 1999 estimates instead of 1998; ^j^ Income share based on 1998 estimates instead of 2000.

### Traditional healer and food costs

Five studies reported that patients paid between I$3 in Malawi [44] to I$563 in Uganda [47] to see traditional healers, and four studies reported the cost of food, which ranged from I$4 (interquartile range (IQR) I$1- I$7) in Zambia to I$36 in Ethiopia and Zambia (median I$19) for “special food” [41,48,58] (see Table 5).

**Table 5:** Traditional healer and food costs

**Author(s) (year)**	**Country**	**Cost estimate (I$)**	**% Annual per-capita income (entire population)**	**% Annual per-capita income (income-poorest 20% of the population)**	**Notes**
**Traditional healer costs**					
Brouwer, et al. [44]	Malawi	4	2	9^a^	Weighted mean of traditional healer costs (range I$0- I$28)^b^
Chard, S. [47]	Uganda	563	188	618^c^	One study participant reported this cost for a traditional healer
Chard, S. [46]	Uganda	15	5	16^c^	Mid-point estimate (range I$2-I$10). A few patients in the sample reported to pay roughly I$495
Needham, et al. [58]	Zambia	17	4	25	Average cost to see a traditional healer (median I$7)
Wyss, et al. [68]	Tanzania	13	4	10^d^	Unit cost to see a traditional healer
**Food costs**					
Aspler, et al. [[41]	Zambia	4	0.78	4	Median food costs (IQR I$1- I$7)
Datiko and Lindtjørn [48]	Ethiopia	14	7	15^e^	Mean food costs for a community-based DOTS treatment program (sd I$12)
Datiko and Lindtjørn [48]	Ethiopia	36	17	37^e^	Mean food costs for a health-facility-based DOTS treatment program (sd I$21)
Kemp, et al. [54]	Malawi	7	4	15^f^	Mean food costs for smear-negative patients (median I$2)
Kemp, et al. [54]	Malawi	10	5	21^f^	Mean food costs for smear-positive patients (median I$0)
Needham, et al. [58]	Zambia	3	1	5	Mean food cost (median I$2)
Needham, et al. [58]	Zambia	36	9	53	Mean cost for “special” foods^g^ (median I$19)

^a^ Income share based on 1998 estimates instead of 1995; ^b^43% of patients in the sample who sought care from traditional healers paid no charge, 21% paid under I$0.92, 24% paid between I$0.92 and I$5, 6% paid between I$6 and I$14, and the remaining 6% paid between I$14 and I$28; ^c^ Income share based on 1999 estimates instead of 1998; ^d^ Income share based on 2000 estimates instead of 1996; ^e^ Income share based on 2005 estimates instead of 2006; ^f^ Income share based on 1998 estimates instead of 2000; ^g^ Defined as food separate from normal diets such as potatoes, eggs, meat, fruit, and soft drinks [58].

### Travel costs

Eighteen studies reported travel costs for patients, families, or guardians for single visits or for multiple visits during treatment. Costs ranged from less than I$1 in South Africa for a single health clinic visit [51,63] to I$70 in Ethiopia for pretreatment transportation costs [65] (see Table 6). Travel time also varied from 48 minutes in Cape Town, South Africa [63] and Kampala, Uganda [46] to almost 70 hours in Ethiopia under a health-facility based DOTS system for a single visit [48]. One study from Zambia distinguished between pre and post diagnosis travel costs: pre-diagnosis travel costs were I$3 (IQR I$1- I$7) while post-diagnosis costs were I$11 (IQR I$4- I$29) [41]. In addition to travel costs, one study reported accommodation costs (in Ethiopia) [55].

**Table 6:** Travel costs

**Author(s) year**	**Country**	**Cost estimate (I$)**	**% monthly per-capita income (entire population)**	**% monthly per-capital income (income-poorest 20% of the population)**	**Notes**
Aspler, et al. [41]	Zambia	3	8	43	Median costs for pre-diagnosis (IQR I$1- I$7)
Aspler, et al. [41]	Zambia	12	31	171	Median costs for pill collection visits (IQR I$4- I$29)
Aspler, et al. [41]	Zambia	4	10	57	Median costs for follow-up visits (IQR I$2- I$4)
Bevan, E. [43]	Kenya	5	11	35	Daily cost to travel to a designated DOTS center
Cambanis, et al. [45]	Ethiopia	7	47	100^a^	Mean costs for transport to a health facility
Chard, S. [46]	Uganda	3	14	45^b^	Mean transportation costs to a health facility in Kampala
Chard, S. [46]	Uganda	5	21	67^b^	Mean transportation costs to a health facility in Mukono
Datiko and Lindtjorn [48]	Ethiopia	2	14	31^c^	Mean transport costs for a community-based DOTS treatment program (sd I$5)
Datiko and Lindtjorn [48]	Ethiopia	15	88	188^c^	Mean transport costs for a health facility-based DOTS treatment program (sd I$43)
Edginton, et al. [49]	South Africa	3	0.96	7^d^	Mid-point costs for 69% of hospital attendees and 48% of clinic attendees (range I$0.52-I$5)^e^
Floyd, et al. [51]	South Africa	12	4	20^f^	Mean travel cost for a hospital visit
Floyd, et al. [51]	South Africa	2	0.74	4^f^	Mean travel cost for a health clinic visit
Floyd, et al. [51]	South Africa	0.17	0.05	0.30^f^	Mean travel cost for a health clinic DOTS visit
Floyd, et al. [51]	South Africa	0.85	0.27	1^f^	Mean travel cost for a TB ward DOTS visit
Floyd, et al. [50]	Malawi	4	26	102	Mean costs for visit to a health center to collect drugs for smear-positive and -negative patients under hospital and community-based strategies (I$18 for average 5 visits)
Harper, et al. [53]	The Gambia	0.55	2	8^g^	Mean daily fare to attend a TB clinic (range I$0.44-I$0.66)
Kemp, et al. [54]	Malawi	18	116	456^h^	Mean transport costs for smear-positive patients (median I$11)
Kemp, et al. [54]	Malawi	13	81	319^h^	Mean transport costs for smear-negative patients (median I$5)
Mesfin, et al. [55]	Ethiopia	11	72	155	Mean transport costs for visiting a public health facility pre-diagnosis
Needham, et al. [58]	Zambia	9	26	150	Mean transportation cost during treatment (median I$3)
Nganda, et al. [60]	Kenya	9	20	67^i^	Mean cost for a visit to collect drugs from a health facility for smear-positive patients under conventional and community-based strategies for smear-positive patients (I$44 for average 5 visits)^j^
Okello, et al. [61]	Uganda	6	24	78^k^	Mean costs to the nearest health facility in an outpatient system and costs to collect drugs under the conventional hospital-based care strategy and the community-based care strategy for smear-positive patients (I$37 for average 5 visits)
Sinanovic, et al. [63]	South Africa	0.40	0.13	0.69^m^	Mean cost for monitoring and collection of drugs and a clinic-based DOTS visit in Guguletu, Cape Town (95% CI I$0.20- I$0.60)
Sinanovic, et al. [63]	South Africa	0.30	0.09	0.52^m^	Mean cost for monitoring and collection of drugs and a clinic-based DOTS visit in Nyanga, Cape Town (95% CI I$0.10- I$0.50)
Vassall, et al. [65]	Ethiopia	70	444	952	Mean pretreatment transportation costs (median I$4)^n^
Wilkinson, et al. [67]	South Africa	5	2	9 ^o^	Average cost of a visit to a village clinic^p^
Wilkinson, et al. [67]	South Africa	20	6	34 ^o^	Average cost of a visit to a hospital^p^
Wilkinson, et al. [67]	South Africa	1	0.43	2 ^o^	Average cost of a village clinic DOTS visit, a community health worker DOTS visit, and a non-health worker DOTS visit^p^
Wyss, et al. [68]	Tanzania	9	32	83 ^q^	Weekly transportation costs

^a^ Income share based on 2005 estimates instead of 2004; ^b^ Income share based on 1999 estimates instead of 1998; ^c^ Income share based on 2005 estimates instead of 2006; ^d^ Income share based on 1993 estimates instead of 1994; ^e^ 29% of hospital attendees and 52% of clinic attendees reported to pay no cost in transportation fees, and 2% of hospital attendees and 0 clinic attendees paid more than I$5; ^f^ Income share based on 1995 estimates instead of 1996; ^g^ Income share based on 1998 estimates instead of 2000; ^h^ Income share based on 1998 estimates instead of 2000; ^i^ Income share based on 1997 estimates instead of 1998; ^j^ The conventional was approach used until 1997 in which new patients were hospitalized for the first month of treatment and subsequently provided unsupervised treatment for the next 11 months. The community-based approach was used after 1997 in which patients spent the first 2 months of treatment in DOTS outpatient programs and the remaining 6 months of treatment in unsupervised outpatient visits; ^k^ Income share based on 1999 estimates instead of 1998; ^l^ Income share based on 2004 estimates instead of 2005; ^m^ Income share based on 1995 estimates instead of 1997; ^n^ Note: these costs are over several months—the authors did not report single transportation costs; ^o^ Income share based on 1995 estimates instead of 1996; ^p^ Note: these costs include indirect time costs; ^q^ Income share based on 2000 estimates instead of 1996.

### Time costs

Twenty-one studies reported time costs. Clinic visit wait time varied from 30 minutes in Limpopo Province, South Africa [49] to 111 minutes in Kampala, Uganda [46]. One study from Uganda reported that patients spent on average 22 minutes for a volunteer-supervised outpatient DOTS visit or I$0.23 (95% CI I$0.00- I$0.42) in lost income, and an average of 110 minutes for a health-facility visit, or I$1 (95% CI I$1- I$2) in lost income [61].

Lost work time varied by treatment system. For hospitalized patients in South Africa, each hospital day led to an average of 402 minutes of lost work time (I$7 in lost income) compared to 128 minutes (I$2) for a health clinic visit, 50 minutes (I$0.85) for a DOTS visit with a community health worker chosen as a treatment supervisor, and 4 minutes (I$0.51) with another type of health worker chosen as a supervisor [51]. In Malawi patients lost an average of 22 workdays resulting in an average income loss of I$68 [54]. Two studies in Zambia found that patients missed an average of 18 workdays before being diagnosed with TB [58] and 48 days of missed work in total [59]. Foregone earnings reported for any type of care-seeking activity ranged from I$3 in South Africa [63] to I$169 in Tanzania [68].

### Reported impact on income

Five studies surveyed patients on their salaries and reported the impact of TB patient costs on household incomes [41,55,58,59,65]; one study used average household income estimates from an external source to calculate the impact of TB patient costs on household incomes [54]. In Malawi, patients spent between 129% and 244% of their mean monthly income (MMI) on TB diagnosis [54]. In Zambia, patients spent 16% of their MMI on transportation costs and 66% of their MMI on food [59]. Direct medical expenditures ranged from between 10% of MMI for men and 132% of MMI for women in Zambia [41] to 31% for all patients in Ethiopia [55], while non-medical expenditures ranged from 42% in Ethiopia [55] to 55% of MMI in Zambia [58]. In Ethiopia, 48% and 35% of annual household income was lost due to TB treatment and pretreatment costs, respectively [65].

### Reported direct, indirect and total costs

Eight studies reported aggregated overall direct costs, and six studies reported aggregated overall indirect costs incurred by patients (although authors defined direct and indirect costs differently). Reported direct costs ranged from I$11 in Zambia [41] to over I$527 in Ethiopia [65], while indirect costs ranged from I$21 in Zambia [41] to I$145 in Ethiopia [55]. Thirteen studies reported overall total costs (direct and indirect), which ranged from I$2 in South Africa [63] to I$584 in Uganda [31] (see Table 7).

**Table 7:** Reported direct, indirect and total costs

**Author(s) year**	**Country**	**Cost estimate (I$)**	**% Annual per-capita income (entire population)**	**% Annual per-capita income (income-poorest 20% of the population)**	**Notes**
**Direct costs**					
Aspler, et al. [41]	Zambia	11	2	13	Total direct costs including medical and non-medical costs (IQR I$6- I$17)
Datiko and Lindtjorn [48]	Ethiopia	17	8	17	Direct patient costs under community-based DOTS (sd I$12)
Datiko and Lindtjorn [48]	Ethiopia	49	24	51	Direct patient costs under health facility-based DOTS (sd I$44)
Kemp, et al. [54]	Malawi	39	21	83^a^	Mean total direct costs for smear-positive patients (median I$19)
Kemp, et al. [54]	Malawi	74	40	156^a^	Mean total direct costs for smear-negative patients (median I$38)
Mesfin, et al. [55]	Ethiopia	114	60	129	Mean total direct costs (median I$61; IQR I$26- I$132)
Needham, et al. [57]	Zambia	64	16	73	Total mean direct costs
Needham, et al. [58]	Zambia	14	3	20	Total mean direct medical costs (median I$5)
Needham, et al. [58]	Zambia	31	8	45	Total mean direct non-medical costs (median I$14)
Vassall, et al. [65]	Ethiopia	527	277	595	Total mean direct pretreatment costs including transport and non-transport costs for (median I$66)
Wandwalo, et al. [66]	Tanzania	59	17	33^b^	Total direct costs under a health facility-based DOTS strategy
Wandwalo, et al. [66]	Tanzania	13	4	8^b^	Total direct costs under a community-based DOTS strategy
**Indirect Costs**					
Aspler, et al. [41]	Zambia	21	5	25	Median total indirect costs (IQR I$11- I$39)
Datiko and Lindtjorn [48]	Ethiopia	18	9	18	Mean indirect cost under community-based DOTS
Datiko and Lindtjorn [48]	Ethiopia	48	50	24	Mean indirect cost under health facility-based DOTS
Mesfin, et al. [55]	Ethiopia	145	76	164	Average indirect costs from first consultation to diagnosis including income lost and travel time cost (median I$44; IQR I$15- I$101)
Mesfin, et al. [55]	Ethiopia	54	28	60	Average indirect costs prior to diagnosis (median I$26.) including income last and travel time cost
Needham, et al. [58]	Zambia	99	25	145	Total lost income (median I$37)
Vassall, et al. [65]	Ethiopia	44	23	50	Total mean indirect pretreatment costs (median I$0)
Wandwalo, et al. [66]	Tanzania	56	16	32^b^	Total indirect costs under a health facility-based DOTS strategy
Wandwalo, et al. [66]	Tanzania	19	5	11^b^	Total indirect costs under a community-based DOTS strategy
**Total Costs**					
Aspler, et al. [41]	Zambia	34	7	41	Total median costs per patients (IQR I$19-I$56) in which direct and indirect costs comprised 34% and 62%, respecitvely
Chard, S. [46]	Uganda	25	8	27^c^	Total reported costs for biomedical treatment
Datiko and Lindtjorn [48]	Ethiopia	34	17	36^d^	Total patient costs under community-based DOTS (sd I$16)
Datiko and Lindtjorn [48]	Ethiopia	99	48	104^d^	Total patient costs under health facility-based DOTS (sd I$50)
Floyd, et al. [51]	South Africa	155	4	23^e^	Total cost to patients under DOTS
Floyd, et al. [51]	South Africa	461	12	67^e^	Total cost to patients under the conventional system^f^
Gibson and Boillot [52]	Sierra Leone	26	10	33^g^	Total cost for patients under the National Leprosy and TB Control Program
Mesfin, et al. [55]	Ethiopia	259	136	292	Mean total costs (median I$119; IQR I$53- I$242)
Needham, et al. [58]	Zambia	68	17	100	Total patient costs (median I$32)
Saunderson, P.R. [31]	Uganda	584	249	809	Total cost under the strategy that utilizes hospitalization for the first two months of treatment followed by an outpatient continuation phase for 4–10 months.
Sinanovic and Kumaranayake [64]	South Africa	102	3	17^h^	Total cost per patient attending a public-non-governmental organization partnership site (95% CI I$73- I$123)
Sinanovic and Kumaranayake [64]	South Africa	95	2	16^h^	Total cost per patient attending a public-non-governmental organization partnership site (95% CI I$82- 104)
Sinanovic and Kumaranayake [64]	South Africa	264	7	44^h^	Total cost per patient attending a public hospital (95% CI I$251- I$274)
Sinanovic and Kumaranayake [64]	South Africa	317	8	53^h^	Total cost per patient attending a public hospital (95% CI I$293- I$363)
Sinanovic, et al. [63]	South Africa	2	0.044	0.24^i^	Total cost for a clinic DOTS visit, where clinic used for DOTS and total cost for monitoring/collection of drugs in Nyanga (95% CI I$1- I$2)
Sinanovic, et al. [63]	South Africa	2	0.041	0.23^i^	Total cost for a clinic DOTS visit, where clinic used for DOTS and total cost for monitoring/collection of drugs in Guguletu (95% CI I$1- I$2)
Sinanovic, et al. [63]	South Africa	1	0.01	0.08^i^	Total cost for a DOTS visit, where community treatment supporter u used for in Guguletu (95% CI I$1- I$2)
Vassall, et al. [65]	Ethiopia	567	298	639	Total mean pretreatment costs
Wandwalo, et al. [66]	Tanzania	116	32	65^b^	Total costs under a health facility-based DOTS strategy
Wandwalo, et al. [66]	Tanzania	32	9	18^b^	Total costs under a community-based DOTS strategy
Wilkinson, et al. [67]	South Africa	183	5	27^j^	Total costs for patients treated under community-based DOTS strategy.

^a^ Income share based on 1998 estimates instead of 2000; ^b^ Income share based on 2000 estimates instead of 2005; ^c^ Income share based on 1999 estimates instead of 1998; ^d^ Income share based on 2005 estimates instead of 2006; ^e^ Income share based on 1995 estimates instead of 1996; ^f^ The conventional system hospitalizes patients for the first two months of treatment; ^g^ Income share assumed to be 6% by authors in absence of World Bank data; ^h^ Income share based on 2000 estimates instead of 2001; ^i^ Income share based on 1995 estimates instead of 1997; ^j^ Income share based on 1995 estimates instead of 1996; ^k^ Income share based on 2000 estimates instead of 1996.

Five studies reported the percentage of all costs that patients paid out-of-pocket. In South Africa, out-of-pocket expenses varied by district in which patients were responsible for paying between 13% and 34% of all costs [51]. Similarly, in Tanzania patients paid between 13% and 30% of total costs in community-based DOTS and health facility-based DOTS programs, respectively [66]. In Malawi and Ethiopia, patients paid close to 50% of total costs of their care.

### Caregiver and guardian costs

Eight studies reported both direct and indirect costs incurred by TB patients’ guardians or caregivers. Direct costs included transportation costs that ranged from less than I$1 (standard deviation [SD] I$4) under a community-based DOTS program in Ethiopia [48] to I$27 in Botswana [56]. The amount of time spent traveling for one care-related visit ranged from 20 minutes in Botswana [56] to 17 hours (median 0) in Ethiopia [55]. Other direct costs for caregivers included food costs that ranged from I$3 (standard deviation [SD] I$5) in Ethiopia [48] to I$1,209 in Botswana [56] and time spent providing care-giving activities, which ranged from 1 hour each day in Botswana [59] to 6 days (median 1) for hospitalized patients in Ethiopia [55]. Indirect costs included foregone earnings for caregivers which ranged from I$19 (median $10) in Zambia [58] to I$89 in Ethiopia [55]. Total reported caregiver cost ranged from I$24 (median I$12) in Zambia [58] to I$1510 under a home-based care strategy in Botswana [56].

### Catastrophic costs

Twenty studies reported costs that were found to be catastrophic for those with average income, and twenty-five studies had costs that were catastrophic for the lowest income earners (see Table 8). Catastrophic costs constituted between 11% of average annual income in The Gambia for private providers [53] and almost three times average annual income in Ethiopia for total pretreatment costs [65]. For those in the income-poorest 20% of a country’s population, catastrophic costs constituted between 10% of annual income for traditional healers in Tanzania [68] to roughly ten times annual income for hospitalization costs in Malawi [50].

**Table 8:** Types of catastrophic costs

**Author(s) (year)**	**Country**	**Catastrophic Costs for Average Income Earners**	**Range (% of annual income)**	**Catastrophic Costs for Lowest 20%**	**Range (% of annual income)**
Aspler, et al. (1998) [41]	Zambia	Total	12	Total, direct, indirect, pre-diagnosis, treatment, time, transportation, medication, hospitalization, direct clinic-based DOTS, indirect clinic-based DOTS	12-40
Awofeso, N. (1998) [42]	Nigeria	Medication	121	Medication	589
Bevan, E. (1997) [43]	Kenya	___	___	Medication, syringes and needles	11-32
Chard, S. (2001) [46]	Uganda	Medication, traditional healer, “tipping” providers	12-165	Medication, traditional healer, “tipping” providers	41-544
Chard, S. (2009) [47]	Uganda	Private provider, traditional healer	47-188	Private provider, traditional healer	154-618
Datiko and Lindtjørn (2010) [48]	Ethiopia	Total, travel, time, caregiver, direct, food	17-48	Total, travel, time, caregiver, direct, food	15-104
Floyd, et al. (2003) [50]	Malawi	Travel, hospitalization, DOTS visit	13-262	Travel, hospitalization	18-1043
Floyd, et al. (1997) [51]	South Africa	Total, hospitalization	11-12	Total, hospitalization	17-67
Gibson, et al. (1998) [52]	Sierra Leone	pre-program, program, hospital admission fees	17-88	Total, pre-program, program, hospital admission fees	32-287
Harper, et al. (2003) [53]	The Gambia	Private treatment	11	Private treatment	54
Kemp, et al. (2007) [54]	Malawi	Direct, income lost, user fees and medication, pre-diagnosis	21-40	Direct, income lost, user fees and medication, pre-diagnosis, food, travel	15-155
Mesfin, et al. (2010) [55]	Ethiopia	Medical, non-medical, indirect, direct, medication, caregiver, total	11-136	Medical, non-medical, indirect, direct, medication, caregiver, travel, total	12-292
Moalosi, et al. (2003) [56]	Botswana	Total caregiver costs Caregiver hospitalization Caregiver food and supplies	13-51	Total caregiver costs, caregiver hospitalization, caregiver medication, caregiver food and supplies	29-258
Needham, et al. (1996) [57]	Zambia	Total medical, direct, income lost	16-148	Total medical, total non-medical, direct, income lost	43-688
Needham, et al. (1998) [58]	Zambia	Total, indirect, pre-diagnosis, non-medical, food	11-47	Total, indirect, pre-diagnosis, non-medical, medical, food, diagnostic tests, caregiver, private provider, traditional healer, consultation fees, travel,	15-214
Needham, et al. (2004) [59]	Zambia	___	___	Transportation	11
Nganda, et al. (2003) [60]	Kenya	Hospitalization, travel	33-101	Hospitalization, travel	22-336
Okello, et al. (2003) [61]	Uganda	Hospitalization, travel	12-73	Hospitalization, travel	40-241
Saunderson, P.R. (1995) [31]	Uganda	Total, hospitalization, pre-diagnosis, indirect	34-249	Total, hospitalization, pre-diagnosis, indirect	111-809
Sinanovic, et al. (2003) [63]	South Africa	___	___	Total	11-43
Sinanovic and Kumaranay-ake (2006) [64]	South Africa	___	___	Total, time, travel	11-53
Vassall, et al. (2010) [65]	Ethiopia	Direct, indirect, travel and total pretreatment	23-298	Direct, indirect, travel and total pretreatment	50-639
Wandwalo, et al. (2005) [66]	Tanzania	Total, direct, indirect	16-32	Total, direct, indirect	18-65
Wilkinson, et al. (1997) [67]	South Africa	___	___	Total, hospitalization	20-27
Wyss, et al. (2001) [68]	Tanzania	Private provider	12	Traditional healer, private provider, hospitalization	10-29

Two aspects of our extracted data are important to note in this context: First, we extracted data for a range of different cost categories (Tables 1, 2, 3, 4, 5). For all cost categories, there are at least a few studies reporting catastrophic costs according to our definition both for those with average income and those with the average income among the income-poorest 20% of the population: traditional healer, food, travel, private provider, medication, “tipping” providers, hospitalization, caregiver, and overall direct, indirect and total costs. Second, the extracted costs are presented in the tables in the units they were reported in the original papers because we did not have sufficient information, either from the papers or external sources, to allow translation into a common unit. However, catastrophic costs were found in all units of reported costs, including per-visit, per time period, and per treatment course and in both the pre- and post-diagnosis periods.

## Discussion

In expanding on a previous review of TB patient costs in sub-Saharan Africa [23], we extended the evidence base on TB patient costs in sub-Saharan Africa through screening of additional databases and broadening the study design inclusion criteria. We have further added to the literature by systematically identifying the particular types of costs TB patient incur and by systematically reviewing the evidence on the cost quantities for each cost type. Our review furthermore provides benchmarks for the magnitude of these cost burdens by comparing them to average income earners and the average income of the income-poorest 20% of the population.

The data reviewed here demonstrate that direct and indirect patient costs for TB patients and their households can be substantial and often “catastrophic” for average income earners and, in particular, for those in the income-poorest 20% of the population—the proportion of the population most at-risk of acquiring TB. The data we extracted from the literature thus suggest that expenditures for TB treatment and care can cause or exacerbate poverty. TB patients in sub-Saharan Africa incur both substantial direct and indirect costs before, during, and after a TB diagnosis. The largest costs these patients incur are for hospitalization, medication, transportation, and treatment or care in the private sector. In addition, caregivers incur substantial indirect, or time costs, of providing care or support for TB patients.

Results also show that total TB treatment and care costs vary greatly between studies: from only I$2 in South Africa [63] to almost $600 in Uganda [31] in total estimated costs. Additionally, the types of costs that patients often pay are numerous: eleven main categories of costs were captured in this review, and nine of the thirty studies reported costs in at least five of these cost categories. Though patients in sub-Saharan Africa often do not incur all of these fees, merely paying for one or some of them can have substantial impacts on their economic circumstances.

Patient costs may negatively impact health-seeking behaviors, leading to delays in hospital presentation, further worsening of disease and increasing risk of disease spread [16]. Patients adopt several mechanisms to cope with these costs ranging from asset selling, borrowing, and diversifying income-generating activities [19,20]. In order to alleviate the financial burden borne by TB patients, policy makers should consider incorporating policies to support patients receiving TB treatment into general financing and risk-pooling strategies, such as tax-based or social insurance systems as used by many developed and, increasingly, developing economies. While in some settings strategies aimed at reducing patient costs incurred when utilizing healthcare may be feared to lead to increases in demand for healthcare exceeding the underlying need, in many developing countries like those in sub-Saharan Africa, healthcare demand is currently far below need, including for the priority diseases TB and HIV, so that financial and non-financial support for healthcare seeking is likely to contribute substantially to improving population health [70,71]. In addition to the direct benefits to the treated patient, TB treatment also reduces onward transmission of the disease in the community. This positive externality needs to be taken into account when considering public investments to decrease the costs patients bare when utilizing TB care.

Comparable types and extents of costs incurred by patients in sub-Saharan have been found in studies assessing the household financial burden of other infectious diseases including malaria and HIV/AIDS. Although some health districts in sub-Saharan Africa provide free care for HIV/AIDS and malaria patients, studies assessing the burden of expenditures for malaria and HIV/AIDS-affected households have found that costs are commonly “catastrophic,” a general finding that conforms with our results for the case of TB. Moreover, the main types of costs that patients and households incurred based on these studies were similar to those found in our review: direct costs of transportation for patients and caregivers, medications, diagnostic tests, hospitalization, food, medical consultations, and traditional healers, as well as the indirect costs such as loss of time at work or school [34,72-76].

More research needs to be done in order to assess the burden of costs to TB patients in other geographical areas, including in the 36 other countries in sub-Saharan Africa for which our systematic review could not identify any empirical estimates of TB treatment and care costs. While our results broadly support calls for health policy changes to alleviate patients’ financial burdens of TB treatment, it is unclear which particular interventions will be most effective and cost-effective. Examples of interventions that can reduce the direct patient expenditures for TB treatment include free transport between communities and TB treatment facilities, food vouchers given to TB patients when visiting a facility, and systematic elimination of user fees. Interventions that can reduce indirect patient expenditures for TB treatment include improved health infrastructure, such as, additional TB treatment facilities, better transport infrastructure (to decrease the time spent travelling from a household to a healthcare facility), as well as improved patient scheduling systems (to decrease patient wait times at healthcare facilities).

Finally, since we find that patients in some countries spend substantial amounts of money for TB treatment and care in the private sector despite public-sector TB programs that offer some services free of charge, interventions to decrease the demand for private-sector TB treatment and care could reduce the financial burden to TB patients. Such interventions could improve media campaigns to ensure that patients are aware of where and when they can access public-sector TB treatment and health sector reforms leading to increased quality of care in the public sector. Rigorous evaluation studies, accompanying the implementation of such interventions, will contribute to our understanding of their performance and impact on patient healthcare expenditures and population health outcomes.

### Limitations

There are several limitations of this review: First, we included studies that were conducted from 1994 to present. Earlier studies might not be generalizable to current strategies because TB control program policies may have changed since their publication. Second, although no official criteria exist for assessing studies on patient costs, other systematic reviews of patient costs have used various quality assessment criteria for evaluating study quality [23,77-79]. Due to our broader study inclusion criteria, we were unable to adopt similar formal criteria for evaluating study quality beyond ensuring that studies had clearly defined objectives, clearly defined study populations, and a quantitative measure of patient-costs. This limitation may have led to a relatively higher weight placed on results from “low quality” studies in the interpretation and discussion of the summary findings from our systematic review than would have been the case if we could have more clearly distinguished between “low” and “high quality” studies.

Third, many studies assessed only a few specific types of costs, so comparing total cost burden to patients across studies is difficult and may be underestimated. Similarly, the studies that reported aggregate direct and indirect costs might have defined direct and indirect costs differently, so these reported measures might not be comparable. Additionally, since costs were measured differently and may reflect different time periods, (for instance some travel costs were measured as one-time costs, and others were measured over the entire course of treatment), comparing these cost burdens on patients’ average annual or monthly income are difficult and must be interpreted with caution.

Moreover, only five studies directly surveyed patients on their incomes. In our estimation of the impact of TB patient costs on patients’ economic circumstances, we thus had to rely on national income estimates. However, it is very likely that TB patients’ average income deviates substantially from the national average, so that our relative measures of the out-of-pocket cost burden to patients and households may be biased.

Furthermore, since data on income shares of the income-poorest 20% of the population were missing for several country-years, we used estimates from the closest years for which data were available for a country, but this could potentially yield inaccurate estimates of per capita GDP for this portion of the population. Finally, evidence on TB patient costs was only available for 11 out of 47 countries in sub-Saharan Africa. Results from these studies cannot be extrapolated to the rest of the continent, and it is possible that patients in other parts of Africa might incur lower TB patient costs.

## Conclusion

Tuberculosis can place considerable financial and economic burden on patients and households in sub-Saharan Africa. Here, we identified 30 relevant studies investigating TB patient costs in sub-Saharan Africa before, during and after diagnosis and treatment for TB. We found that patient costs vary considerably both by total amounts and according to the different types of costs incurred by patients in DOTS and non-DOTS settings. In many settings, patient costs were found to be “catastrophic” in that they amounted to 10% or more of average annual incomes. These costs have the potential to financially strain patients and their households, leading to detrimental effects such as delayed care seeking and increased default rates of TB patients, potentially fueling the spread of TB or increasing multi-drug resistant TB in sub-Saharan Africa. Future research needs to assess which interventions and health systems reforms are most effective and cost-effective in reducing the financial burdens that patients incur when seeking TB treatment and care.

## Competing interests

The authors declare that they have no competing interests.

## Authors’ contributions

DB designed the study, conducted the search, extracted and interpreted data, and wrote the manuscript. SA served as a second reviewer for article inclusion and exclusion, extracted data and assisted with the manuscript. TB and MB supervised the study and contributed to study design, methodologies, interpreted results, and assisted with writing the final manuscript. All authors read and approved the final manuscript.

## Pre-publication history

The pre-publication history for this paper can be accessed here:

http://www.biomedcentral.com/1471-2458/12/980/prepub

## Supplementary Material

Additional file 1 Systematic review search algorithm. Click here for file
