# Supplementary material for: Tuberculosis and poverty: the contribution of patient costs in sub-Saharan Africa – a systematic review
Source: BMC Public Health. 2012 Nov 14;12:980. doi: 10.1186/1471-2458-12-980 (PMC3570447; doi:10.1186/1471-2458-12-980)
Supplement: Additional file 1 — Systematic review search algorithm. [file 1471-2458-12-980-S1.docx]

**Additional Files**

**Systematic review search algorithm**

**PubMed**

("tuberculosis"[mesh] OR ("tuberculosis"[MeSH Terms] OR "tuberculosis"[All Fields]) OR TB[all fields])

AND

("Employment"[MeSH Terms] OR "employment"[all fields] OR "out of pocket"[All Fields] OR "direct costs"[All Fields] OR "indirect costs"[All Fields] OR "patient cost"[All Fields] OR "Costs and Cost Analysis"[Mesh])

AND

1945/01[PDAT] : 2011/01[PDAT]

**EMBASE**

'cost'/exp OR 'cost of illness':ab,ti OR 'indirect cost':ab,ti OR 'direct cost':ab,ti OR 'out-of-pocket':ab,ti OR 'patient cost':ab,ti

AND

('tuberculosis'/exp OR 'tuberculosis')

AND

[1-1-1945]/sd NOT [1-1-2011]/sd

**Science Citation Index and Social Science Citation Index**

Topic=(TS=(cost* SAME tuberculosis)) [date range: 1900-2010]

**EconLit**

(cost* or employment or income)

AND

KW=(tuberculosis or TB) [Specified date range “earliest to 2010”]

**Dissertation Abstracts**

(TB) OR (tuberculosis )

AND

(cost*) [date range: 1900 -2010]

**CINAHL**

(MH "Economic Aspects of Illness") or cost*

AND

((MH "Tuberculosis ")) [Limiters - Published Date from: 19830101-20101231]

**Sociological Abstracts**

 ((socioeconomic or employment or income) or (cost* or out-of-pocket or expense*))

AND

(DE=tuberculosis)
